# Acute and chronic gene expression activation following medial forebrain bundle DBS and selective dopamine pathway stimulation

**DOI:** 10.1038/s41598-025-91994-x

**Published:** 2025-02-28

**Authors:** Zhuo Duan, Wen Zhao, Yixin Tong, Volker A. Coenen, Máté D. Döbrössy

**Affiliations:** 1 Laboratory of Stereotaxy and Interventional Neurosciences, Department of Stereotactic and Functional Neurosurgery, University Freiburg - Medical Centre, Breisacher Str. 64, 79106 Freiburg, Germany; 2 Department of Stereotactic and Functional Neurosurgery, University Freiburg - Medical Centre, Freiburg, Germany; 3 Faculty of Biology, University of Freiburg, Freiburg, Germany; 4 Faculty of Medicine, University of Freiburg, Freiburg, Germany

**Keywords:** Biological techniques, Molecular biology, Neuroscience

## Abstract

Deep brain stimulation (DBS) of the medial forebrain bundle (mfb) demonstrated anti-depressant effects both clinically and experimentally. Modulation of mesocorticolimbic dopaminergic (DA) activity could contribute—in part—to the therapeutic effects. By comparing selective and pathway specific midbrain DA optogenetic stimulation with the global, non-pathway specific mfb-DBS, the study explored changes in gene-expression of key biomarkers associated with neurocircuitry of depression. Rats received either optogenetic DAergic or mfb-DBS, delivered as acute/single or chronic/repeated stimulation. Micro-dissected regions were prepared for in situ hybridization targeting biomarkers of GABAergic, glutamatergic, and dopaminergic systems. Mfb-DBS mediated DA independent pathway increased GABAergic biomarkers (*GABAA*, *GAD1*) in frontal and accumbal regions, not in midbrain. The combinations of low frequency/high pulse width and high frequency/low pulse width stimulation generally increased biomarker expression similarly, but chronic/repetitive stimulation had no accumulative effect. Interestingly, unilateral stimulation had bilateral effects, but stimulation modalities had little impact on *DAT* and *Vglut2* expression. In conclusion, both low and high frequency, acute/single and chronic/repetitive mfb-DBS—but not selective optogenetic stimulation -activated gene expression of biomarkers associated with GABAergic transmission. The increased expression was transitory and less chronic than predicted. Importantly, the study provides evidence that the anti-depressant therapeutic effects of clinical medial forebrain bundle DBS occurs—in part—be via modulation of GABAergic signalling which in turn could regulate the release of dopamine in frontal and accumbal regions. In addition, clinical implication of the data is that unilateral stimulation had bilateral consequences on the gene expression, although the physiological and functional sequelae of this are yet unknown.

**Supplementary Information:**

The online version contains supplementary material available at 10.1038/s41598-025-91994-x.

## Introduction

The prevalence and impact of Major Depressive Disorder (MDD) on global health is significant, with a vast number of individuals suffering from its debilitating effects^1^. Conventional treatments, while effective for some, still leave one third of the patient population in the Treatment-Resistant Depression (TRD) category. Recent advancements in neuromodulation, specifically Deep Brain Stimulation (DBS) of superolateral medial forebrain bundle (in the text “MFB” refers to humans and “mfb” to rodents) demonstrated rapid and long-term clinical efficacy in TRD patients^2–5^. Anti-depressant effects in experimental rodent depression models have also been evidenced^6,7^.

The mfb contains bi-directional, myelinated and unmyelinated projections of numerous neurotransmitter and modulators, including dopamine (DA), glutamate, and GABA^8^. The mfb fibers connect regions associated with functions such as motor, cognition and emotion/mood processing, including components of the “reward” network such as the medial prefrontal cortex (mPFC), the nucleus accumbens (NAc), and the ventral tegmental area (VTA)^9^. The ascending midbrain mesocorticolimbic dopamine projections originate in the VTA and projects via the mfb to the NAc and prefrontal cortex (PFC)^10^. Key symptoms in depression, such as, anhedonia, or reduced motivation, can be explained by dysfunctional DA transmission through the mfb^11^. For example, the selective, excitatory optical stimulation of DA neurons in the VTA^12^ or in the mfb^13^, produces anti-depressant-like effects in the rodent chronic mild stress models and the dopamine release in NAc has been recorded post mfb-DBS^14^. Other than DA, VTA contained also small but significant amount of glutamatergic projections. Recent studies suggest that the glutamate neurons in VTA play a crucial role in a variety of behaviors such as reward reinforcement, aversive behaviors, wakefulness, and defensive behaviors^15–18^. A substantial proportion of neurons that project from the VTA to the NAc and mPFC express *Vglut2*^19–22^, indicating that glutamate release from the VTA to these areas is vital for its modulatory functions. Moreover, VTA glutamatergic pathways extend to regions traditionally not associated with dense dopaminergic innervation including the lateral habenula (LHb) and dorsal hippocampus^23,24^. Involvement of the glutamatergic system are also evidenced by findings that ketamine, an NMDA antagonist, acts at the aversive/anti-reward center LHb and promotes a rapid anti-depressive effect^25^. Similarly, rapid on-set of therapeutic effects have been reported in clinical trials of MFB-DBS^2,3^. The third set of biomarkers studied pertained to GABAergic transmission. GABAA receptor has been implicated in the modulation of anxiety- and “depression-like” behaviors^26^, and GABAergic inhibitory interneurons participate in the mechanisms of action of DBS through the regulation of the activity of VTA dopaminergic neurons^27^.

To shed light on the mechanisms of action of neurostimulation in a novel way, the current study directly compared the biological consequences of acute/single and chronic/repetitive selective midbrain DA optogenetic stimulation (30 Hz/5 ms), and non-selective mfb-DBS, using different frequency and pulse-width stimulation parameters (30 Hz/5 ms; or 130 Hz/100 µs). We hypothesized that changes in gene expression of key biomarkers from the GABAergic, glutamatergic, and dopaminergic systems in neuronal hubs associated with neurocircuitry of depression would affected differentially by the two types of neuromodulation, as well as by the different temporal nature of the stimulation. We report that both low and high frequency, acute/single and chronic/repetitive mfb-DBS—but not selective optogenetic stimulation -activated gene expression of biomarkers associated with GABAergic transmission, but the increased expression were transitory and less chronic then predicted. Furthermore, the data suggests that unilateral stimulation produces bilateral changes in biomarker expression and postulates that transmitter release—although not measured in the current study—can occur without accompanying changes in gene expression.

## Method

### Subjects

Female Long-Evens TH-cre rats and their wild-type littermates from our own colony were used in the experiment. Animals were genotyped on post-natal day 14 and allocated to either the optogenetic stimulation group if they carried the Cre gene (*n* = 20), or into the DBS group if they were wild-type (*n* = 25). Housing conditions for all animals were identical. They were individually housed in Plexiglas cages (40 cm × 40 cm × 40 cm) with *ad libitum* access to food and water. The facility was maintained at a 12:12 light/dark cycle, with ambient conditions of 23 ± 1 °C temperature, 50–60% humidity, and light intensity capped at 60 lx. All animal procedures were carried out in accordance with the ethical standards and the approval of the University of Freiburg (Regierungspräsidium; TVA G14-40) and adhered to the EU Directive 2010/63/EU for animal protection in scientific research, and to the Animals (Scientific Procedures) Act 1986, International Association for Study of Pain^28^ and ARRIVE guidelines^29^.

### Virus injection and stereotactic surgery

Animals underwent anesthesia in an induction chamber with 4% isoflurane and 2 L/min oxygen, before transfer to a stereotactic frame where isoflurane levels were maintained at 1.5–2%. Surgery was performed referencing the “flat skull” position to ensure accurate coordinates. Coordinates for the virus injection/optic fiber implantation^13^ and for the electrode implantation^6,7^ were based on previous experience. For the optogenetic stimulation group, AAV2-Ef1α-DIO-hChR2-EYFP with titers of 4.2 × 10^12^ particles per ml was loaded into a 2 µl Hamilton syringe and administered into the VTA (AP: −5.6 and − 6 mm, ML: −0.7 mm, DV: −7.5 and −8.2 mm) at 100 nl/min^13^. Following the 2000 nl injection, a 10 min waiting period followed to prevent viral backflow before slowly withdrawing the syringe. After injection, the optic fiber was implanted in an angle of 3° at right mfb (AP: −2.8 mm, ML: −2.5 mm, DV: −7.5 mm) with four anchoring screws on the skull close to the implant. Dental cement was added around the screws and the optic fiber to fix the implant to the skull, and further secured by bone cement. For the DBS group, the bipolar electrode (two, 15 mm Teflon coated/isolated, twisted 125 m diameter, 90% platinum/10% iridium electrodes, World Precision Instruments, Sarasota, USA) was inserted into right mfb (AP: −2.8 mm, ML: −1.7 mm, DV: −7.9 mm) with anchoring screw on the skull. After implanting, the electrode was secured by dental cement and assembled to the 10-contacts plug. Bone cement was used to further secure the plug and electrode. Following the implantation surgery (optic fiber or DBS electrodes), animals were individually housed and received pain management with Buprenovet (0.05 mg/kg). Animals were closely monitored for signs of infection or distress.

### Optogenetic stimulation

Post-surgery, the animals were placed in individual cages to protect the implants and were given a week to recover and to habituate to their novel environment. Before commencing with the stimulation protocol, the light intensity was calibrated using a power meter (PM130D, Thorlabs). The animals were then securely coupled to the patch cord via mating sleeves (Doric Lenses) affixed to the head cannula, and placed into a specifically designed stimulation cage with an opaque lid to minimize external light interference. Stimulation parameters were programmed to deliver eight pulses at a 5 ms pulse width and a frequency of 30 Hz^13,30^, with a 5-second interval between each sequence. Rats received either a single 30 min optogenetic stimulation (*n* = 8), or a 30 min a day for 7 days (*n* = 8), while the sham control animals (*n* = 4) received no stimulation. Upon completion, animals were decoupled from the patch cord and subsequently administered terminal anesthesia in preparation for histological examination.

### Deep brain stimulation

Post-surgery, the animals were placed in individual cages to protect the implants and were given a week to recover and to habituate to their novel environment. The stimulation setup included a specifically designed cage, a pulse generator (STG 2008, Multichannel Systems), connecting cables, and a voltage and impedance monitor. Mfb DBS parameters were square-wave biphasic, constant current pulse pairs, commencing at either 30 Hz with a 5 ms pulse width (identical to the optogenetics parameters) or 130 Hz with a 100 µs pulse width as aligned with clinical trial parameters. Ten rats received single 30 min DBS (30 Hz 5 ms, *n* = 6; 130 Hz 100 µs, *n* = 4); 13 rats received DBS for 30 min/day for 7 days (30 Hz 5 ms, *n* = 7; 130 Hz 100 µs, *n* = 6); two rats received no stimulation as sham control. The stimulation intensity was titrated from 50 µA to 350 µA in 50 µA steps. SEEKING behavioral was used as the indicator signaling the appropriate intensity (see^31^ for full description). Throughout the experiment, voltage and impedance were closely monitored to prevent excessive charge that might compromise the brain-electrode interface. After the final DBS session, animals were detached from the system and administered terminal anesthesia for subsequent histological analysis.

### Immunohistochemistry

Animals were administrated a terminal dose of 10% ketamine with 2% xylazine, and perfused by intracardial infusion of phosphate-buffered saline (PBS) and followed by 4% paraformaldehyde. After perfusion, the brains were extracted under RNase-free condition and stored in sucrose for 1 day followed by storage at − 80 °C until sectioning. The brains were cryo-sectioned at 50 μm thick coronal slices and collected into 12 wells. The sections near the implantation site were used for immunohistochemical staining to verify implant location. For the optogenetic stimulated group, after washing in PBS and 1 h blocking by 5% bovine serum albumin with 0.3% triton X-100, the sections at the mfb were stained by anti-GFP (A11120, 1:500, Invitrogen) against the viral reporter gene and at the VTA by anti-TH (PRB515P, 1:600, Covance) to identify the dopaminergic fibers/neurons. Secondary antibodies were Alexa Fluor 488 goat anti-mouse (A11001, 1:200, Life Tech) and Alexa Fluor 568 goat anti-rabbit (A11011, 1:200, Life Tech), respectively. Sections were cover slipped. For the DBS group, sections were incubated overnight with anti-TH primary antibodies (PRB515P, 1:600, Covance) followed by washing and biotinylated anti-rabbit secondary antibodies (E0432, 1:200, Dako) for 1 h at room temperature. Subsequent to additional washes, sections were treated with the avidin-biotin-complex (ABC) solution. Visualization of the antibodies was achieved by incubating the sections in DAB/H_2_O_2_ working substrate solution until the desired staining level was reached, ensuring minimal background staining. Sections were washed in PBS to stop the reaction and cover slipped.

### In-situ hybridization

The sections containing the regions of interests (ROIs) (mPFC, NAC, LHb, SN, VTA, DStr) underwent in-situ hybridization. RNA probes for *DAT*, *VGLUT2*, and *GABAA*, *GAD1* were synthesized via in vitro transcription of cDNA sequences from GenBank. The process involved cloning cDNA into plasmid vectors with SP6, T3, or T7 promoters. Plasmids were cultivated, isolated, and linearized with according restriction enzymes. The linearized DNA served as templates for in vitro transcription using DIG RNA labeling mix, and RNA polymerase, with the reaction mixture subsequently treated with DNase I. RNA probes were precipitated, washed, and their concentrations verified by dot blotting. For hybridization, tissue samples were processed under RNase-free conditions, pre-hybridized, and hybridized with probes in a specifically formulated buffer at 55 °C. Post-hybridization, samples were washed, blocked, and incubated with anti-dig-AP for probe detection. Colorimetric detection was achieved using NBT/BCIP, with varying incubation times per target gene. Finally, samples were washed, stored in PBS, and prepared for analysis.

### Imaging acquisition and quantification

The epifluorescence images were acquired using the Axioscan 7 (Zeiss, Germany) and were corrected for brightness and contrast and exported by Zen 2.5 software. The DAB stained and in-situ hybridization images were digitized with a slide scanner (3D HISTECH Pannoramic DESK II DW) and the respective software Pannoramic Viewer (3DHISTECH, n.d.-c). The images converted with the Slide Convertor (3DTECH) into a suitable TIFF format. An automatic counting script of ImageJ conducted the quantification of positive stained cells with preprocessing by plugin “Bio-formats” and “shape filter”. Specifically, ROIs from mPFC (AP = + 3.0), DStr (AP = + 1.68), NAc (AP = + 1.68), HPC (AP = −3.60), LHb (AP = −3.60), VTA (AP = −5.52), and SN (AP = −5.52) were manually outlined based on the Paxinos rat brain atlas^32^. Images were processed by converting to RGB stacks and then to 8-bit grayscale. They were rotated 90° for correct orientation. To reduce noise, a Gaussian Blur (1 µm radius) was applied. Segmentation was performed using “Auto Local Threshold” with the ”Phansalker” method (radius: 15) to differentiate signal from background. Bubbles were removed using IJBlob’s “Solidity” filter (solidity range: 0.3–1). Finally, ”Watershed” was applied to separate touching particles, ensuring accurate cell counts. Cell counting was automated using ImageJ’s “Analyze Particles” function. Within the selected ROIs, cells were quantified based on a size range of 10 to 700 μm^2^ and a circularity range of 0.20 to 1.00. The resulting data were systematically compiled and saved in an Excel file for further analysis.

### Statistical analysis

Advanced graphing and statistical analyses were carried out using GraphPad Prism. Initially, the Shapiro-Wilk test was utilized to assess data normality. For parametrical data, we employed one-way ANOVA to evaluate statistical differences across experimental groups in our study. For non-parametrical data, a Kruskal-Wallis test was conducted. All data are presented as mean ± SEM, with statistical significance set at *p* < 0.05.

## Results

**Fig. 1:**
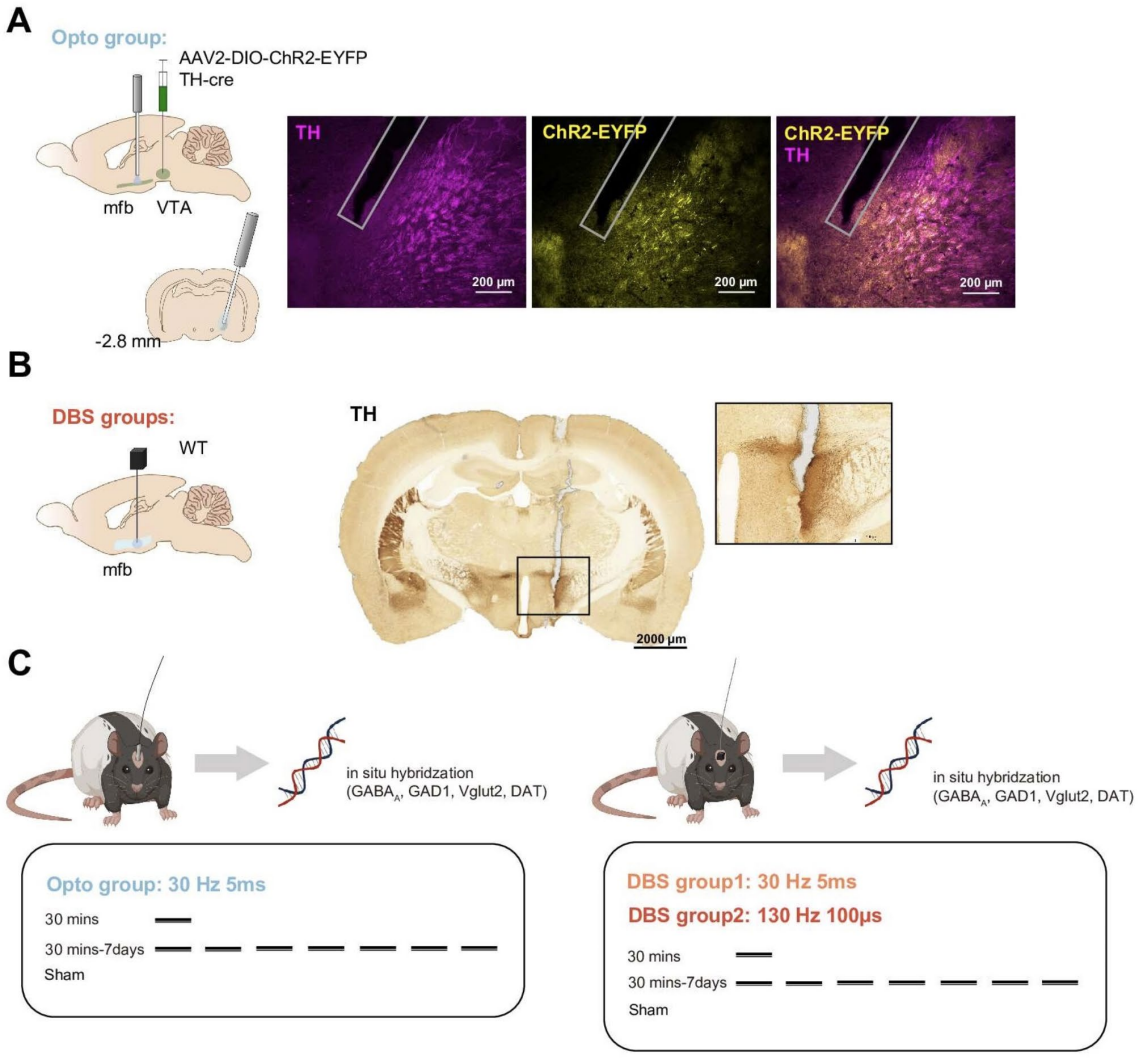
(**A**–**C**) Characterization of the Optogenetic and Deep Brain Stimulation (DBS) Protocols in medial forebrain bundle (mfb). (**A**) Optogenetic group: Representative image showing the expression of AAV2-DIO-ChR2-EYFP in dopaminergic fibers of mfb originating from the ventral tegmental area of TH-cre rat, as indicated by tyrosine hydroxylase (TH) staining. (**B**) DBS groups: Coronal brain section illustrating the placement of the stimulating electrode in the mfb. The inset shows a higher magnification of the electrode track with TH staining to highlight the dopaminergic pathway. (**C**) Experimental timeline and stimulation protocol for both optogenetic and DBS groups.

Changes in the gene expression of biomarkers was studied following mfb stimulation either via selective optogenetic stimulation of the DAergic projections or following non-selective DBS. In the optogenetic group, we targeted reward mediating A10 dopaminergic fibers in the mfb originating from VTA by TH-cre rats. AAV2-DIO-ChR2-EYFP was injected into VTA and the expression in DA neuron axons in mfb were confirmed by TH staining (Fig. 1A). Similarly, DBS groups showed precise electrode placement within the defined mfb^33^ with TH-positive DA fibers along the electrode track in coronal brain sections (Fig. 1B). Two experimental protocols were conducted in between-group design for each conditions including an acute single 30 min stimulation or a 30 min/day stimulation for 7 days. The optogenetic group received 30 Hz stimulation at 5 ms pulse duration; the DBS group received either 30 Hz/5 ms (similarly to the optogenetic group) or 130 Hz/100 µs stimulation (Fig. 1C).

### GABAergic system alterations in depression-related brain regions following Mfb DBS

Changes in GABAergic system were identified by assessing changes in the gene expression of *GABAA* or *GAD1* in key regions associated with depression neurocircuitry, including mPFC, DStr, NAc, HPC, LHb, VTA, and SN. Comparative analysis was conducted across various groups: an unstimulated sham group; a group receiving 30 Hz/5 ms DA dependent optogenetic stimulation, a group with 30 Hz/5 ms non-selective mfb-DBS, and finally, a mfb-DBS group with clinical parameter stimulation of 130 Hz/100 µs.

**Fig. 2:**
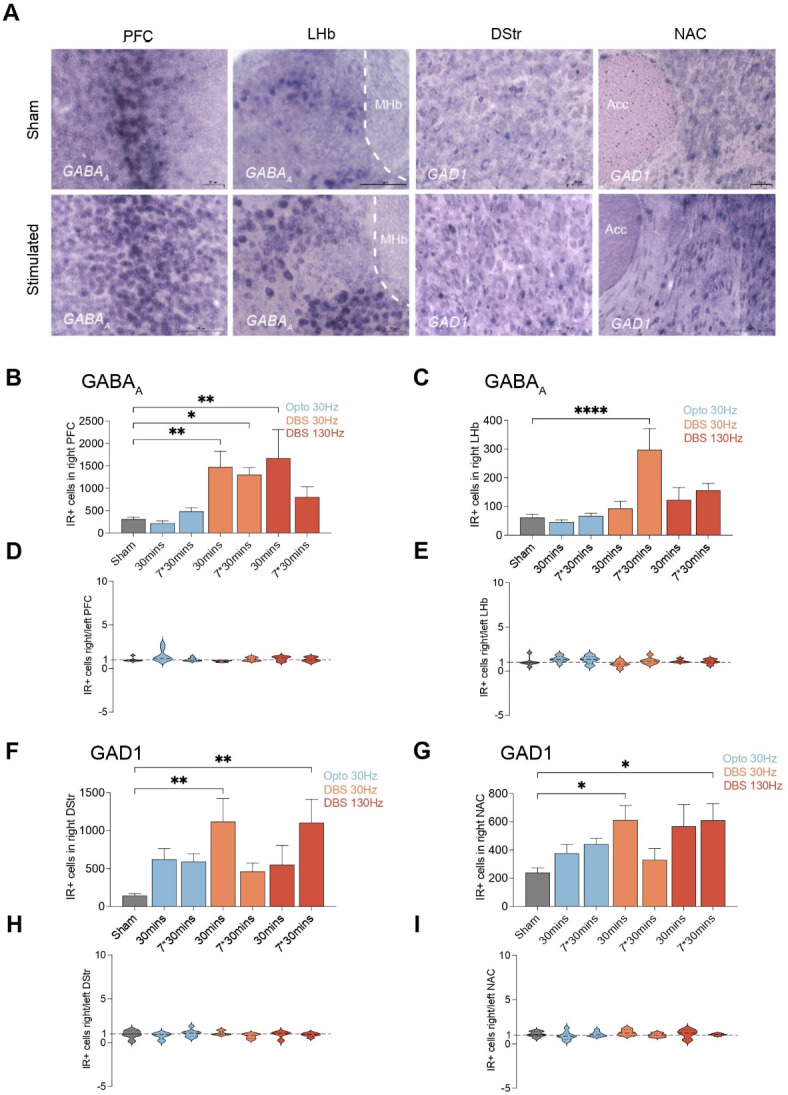
(**A**–**I**). GABAergic systems influenced by optogenetic dopamine dependent medial forebrain bundle (mfb) stimulation and dopamine independent mfb Deep Brain Stimulation (DBS). (**A**) Representative picture of *GABA*_*A*_*/GAD1* gene expression in the medial prefrontal cortex (mPFC), lateral habenula (LHb), dorsal striatum (DStr), and nucleus accumbens (NAC) influenced by mfb-DBS. (**B**) The immunoreactive positive (IR+) cells for *GABA*_*A*_ in right (implanted) mPFC. (**C**) The IR + cells for *GABA*_*A*_ in right (implanted) LHb. (**D**) The ipsilateral/contralateral ratio of IR + cells in mPFC. (E) The ipsilateral/contralateral ratio of IR + cells in LHb. (**F**) The IR + cells for *GAD1* in right (implanted) DStr. (**G**) The IR + cells for *GAD1* in right (implanted) NAC. (**H**) The ipsilateral/contralateral ratio of IR + cells in DStr. (**I**) The ipsilateral/contralateral ratio of IR + cells in NAC. Statistical significance is indicated as follows: *p < 0.05, **p < 0.01, ***p < 0.001, ****p < 0.0001.

**Fig. 3:**
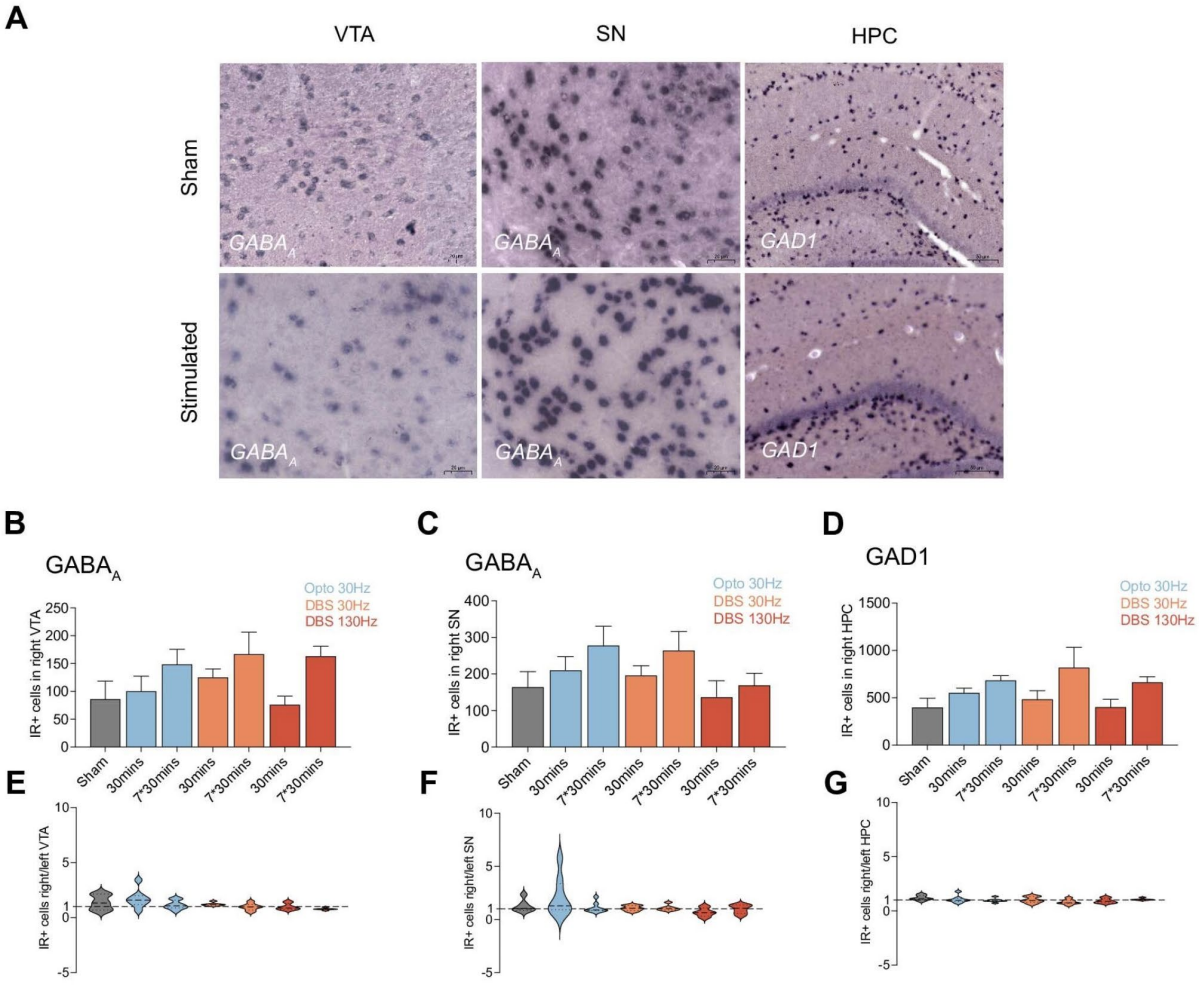
(**A**–**G**) GABAergic systems were not influenced by optogenetic dopamine dependent medial forebrain bundle (mfb) stimulation and dopamine independent mfb Deep Brain Stimulation (DBS). (**A**) Representative picture of GABA_A_/GAD1 expression in the ventral tegmental area (VTA), substantia nigra (SN), and hippocampus (HPC) influenced by mfb-DBS. (**B**) The immunoreactive positive (IR+) cells for *GABA*_*A*_ in right (implanted) VTA. (**C**) the ipsilateral/contralateral ratio of IR + cells in VTA. (**D**) The IR + cells for *GABA*_*A*_ in right (implanted) SN. (**E**) the ipsilateral/contralateral ratio of IR + cells in SN. (F) The IR + cells for *GAD1* in right (implanted) HPC. (**G**) the ipsilateral/contralateral ratio of IR + cells in HPC. Statistical significance is indicated as follows: *p < 0.05, **p < 0.01, ***p < 0.001, ****p < 0.0001.

Our investigation revealed significant changes in the expression of *GABAA* and *GAD1* within the mPFC, LHb, DStr, and NAc following DBS (Fig. 2A).Comparing changes on the implanted ipsilateral side under different stimulation conditions revealed that optogenetically activating DA fibers in the mfb and electrically stimulating the mfb differentially influenced GABAergic expression. In the mPFC, a substantial increase in immunoreactive (IR) positive cells for *GABAA* was observed in the ipsilateral hemisphere where the stimulation was applied between the sham control and several experimental groups ([F(6, 38) = 3.052], *P* < 0.0001). Multiple comparisons indicated that the mean value was significantly different between the sham and the 30 Hz 5 ms DBS group (*P*_30 mins_ = 0.0053, *P*_7*30 mins_ = 0.0166) and the acute 30 min 130 Hz 100 µs DBS group (Fig. 2B; *P* = 0.0033). No statistically significant difference were observed between the sham and the optogenetic group or the chronic 7*30 mins 130 Hz 100 µs DBS group. Similar results were seen in the LHb, with significant differences found between the sham and the chronic 7*30 min 30 Hz 5 ms DBS group (Fig. 2C; *P* < 0.0001). Further analysis in the DStr and NAc showed a significant increase in IR positive cells for *GAD1* (Fig. 2F and G). Multiple comparisons revealed that the mean value was significantly different between the sham and the acute 30 min 30 Hz 5 ms DBS group (*P* = 0.0025) and the chronic 7*30 mins 130 Hz 100 µs DBS group (*P* = 0.0090) in the DStr and the acute 30 min 30 Hz 5 ms DBS group (*P* = 0.0222) or the chronic 7*30 min 130 Hz 100 µs DBS group (*P* = 0.0228) in the NAC, respectively. No significant changes in *GABAA* gene expression were found in the VTA and SN (Fig. 3A–C), nor in *GAD1* gene expression in the HPC (Fig. 3D).

The IR positive cells in both hemispheres were quantified, and the ipsilateral-to-contralateral ratio was calculated to evaluate the lateralization effects of stimulation. However, the ratios maintained consistency across different groups at around one to one ratio, indicating no lateralization and the stimulation produce a bilateral effect (Figs. 2D, E, H and I and 3E, F and G).

### Ascending dopaminergic output respond to Mfb stimulation

**Fig. 4:**
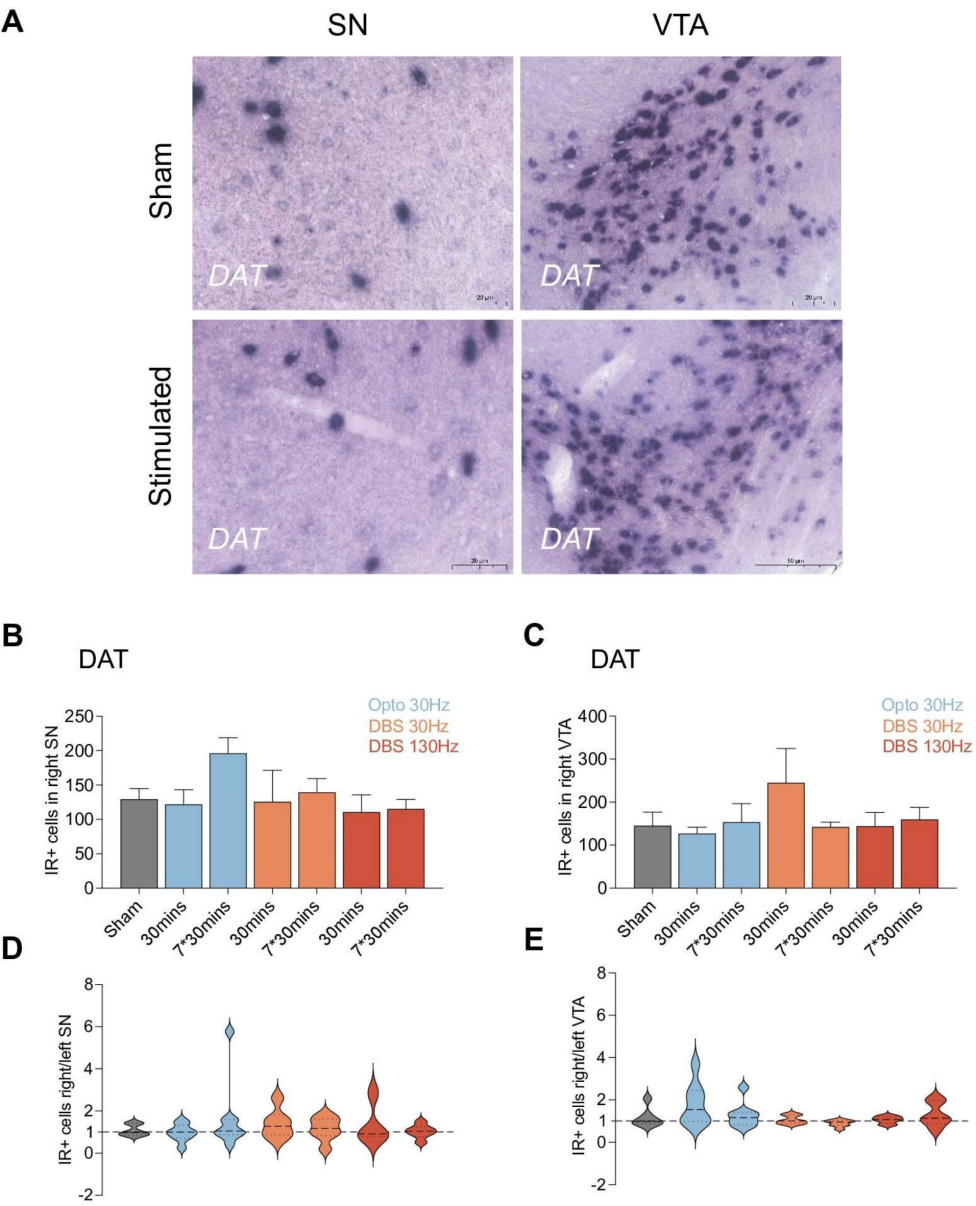
(**A**–**E**) A9 A10 dopaminergic systems were not influenced by optogenetic dopamine dependent medial forebrain bundle (mfb) stimulation and dopamine independent mfb Deep Brain Stimulation (DBS). (**A**) Representative picture of *DAT* expression in the ventral tegmental area (VTA) and substantia nigra (SN). (**B**) The immunoreactive positive (IR+) cells for *DAT* in right (implanted side) VTA. (**C**) The IR + cells for *DAT* in right (implanted side) SN. (D) the ipsilateral/contralateral ratio of IR + cells in SN. (E) the ipsilateral/contralateral ratio of IR + cells in VTA. Statistical significance is indicated as follows: *p < 0.05, **p < 0.01, ***p < 0.001, ****p < 0.0001.

Midbrain dopaminergic neurons were identified by the *DAT* gene marker, and stimulation evoked changes in its expression were assessed in VTA (A10) and SN (A9). No significant up or down-regulation of the *DAT* gene expression were observed across the different experimental conditions (Fig. 4A). Comparing the effects of each stimulation condition against a sham intervention corroborated these findings, showing no significant differences in *DAT* expression within either the VTA (Fig. 4B) or SN (Fig. 4C). Further, we quantified IR positive cells in both hemispheres and computed the right-to-left ratio as a measure of the lateralization impact of the stimulations. The ratios remained consistent across different experimental groups, with an average close to 1.0, indicating that the unilateral stimulation evoked similar effects bilaterally (Fig. 4D and E).

### Glutamatergic alternation of reward and anti-reward center after Mfb DBS

**Fig. 5:**
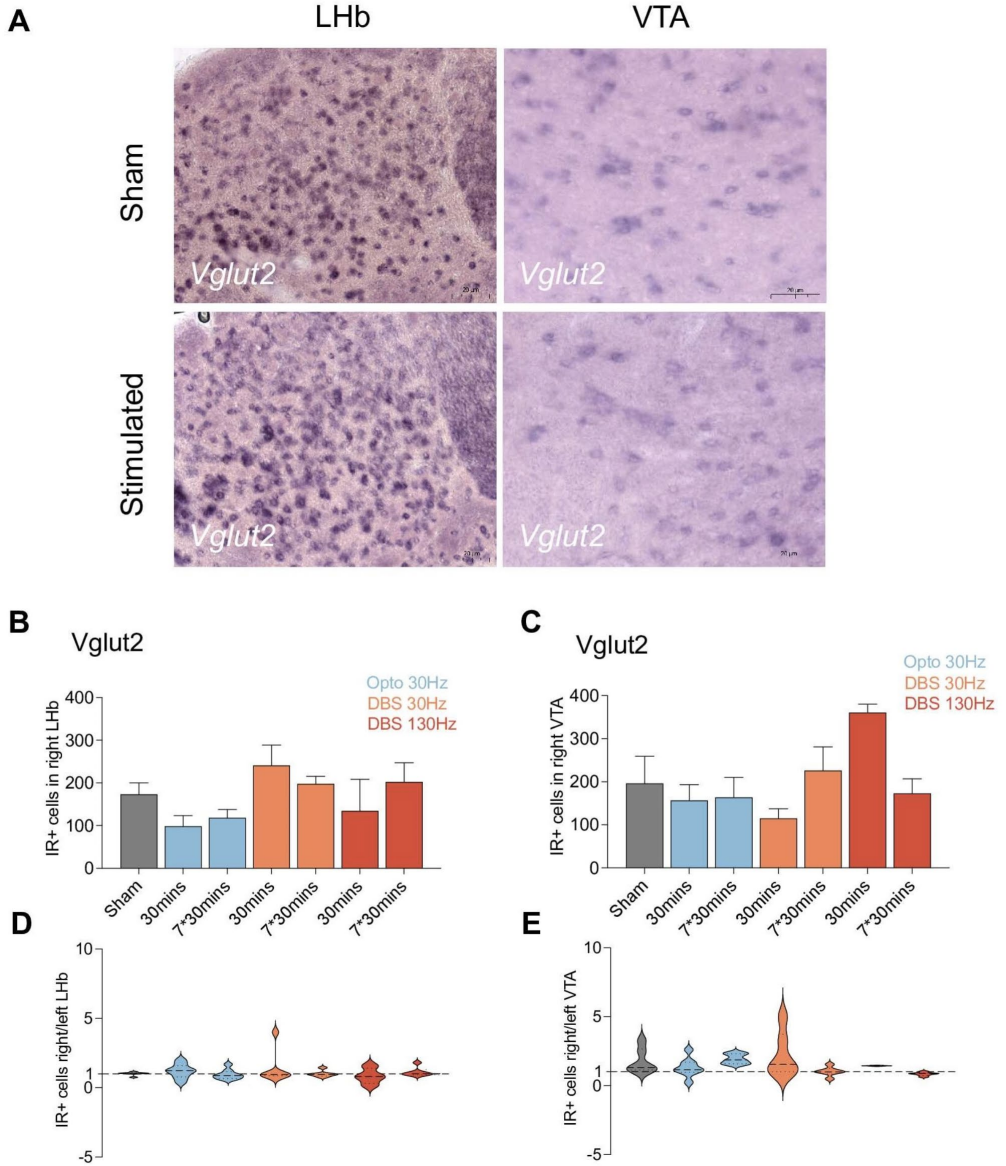
(**A**–**E**) Reward and antireward center glutamatergic systems were not influenced by optogenetic dopamine dependent medial forebrain bundle (mfb) stimulation and dopamine independent mfb Deep Brain Stimulation (DBS). (**A**) Representative picture of Vglu2 expression in the lateral habenula (LHb) and ventral tegmental area (VTA). (**B**) The immunoreactive positive (IR+) cells for Vglu2 in right (implanted) LHb. (**C**) The IR + cells for Vglu2 in right (implanted) VTA. (**D**) The ipsilateral/contralateral ratio of IR + cells in LHb. (**E**) the ipsilateral/contralateral ratio of IR + cells in VTA. Statistical significance is indicated as follows: *p < 0.05, **p < 0.01, ***p < 0.001, ****p < 0.0001.

Glutamatergic neurons were identified by the *Vglut2* gene marker coding for glutamate transporter (Fig. 5A). Changes in the expression of *Vglut2* gene expression in a key hub of the reward center, the VTA, and in the anti-reward center, the LHb, were quantified in the different experimental stimulation groups. omparing unstimulated sham controls with optogenetically and DBS stimulated groups were conducted for VTA and LHb, respectively. The results indicated no significant differences in the number of IR positive neurons between either the optogenetic or DBS groups under any stimulation parameters in neither the VTA (Fig. 5B) and LHb (Fig. 5C).

The number of IR positive neurons in both hemispheres was quantified. The ipsilateral-to-contralateral ratio was calculated to assess potential lateralization effects of the stimulations. These ratios remained consistent across the different groups, with an average close to 1.0, suggesting no lateralization effect due to stimulation. The stimulations appeared no influence in glutamatergic neuron (VTA and LHb) of both hemisphere, as no lateralization was detected (Fig. 5D and E).

## Discussion

Optogenetic and electrical stimulation of the medial forebrain bundle (mfb) have shown to produce dopamine release in the nucleus accumbens or the prefrontal cortex in experimental models^34–37^. The current study investigated changes in biomarker expression—evoked by general electrical mfb-DBS or selective midbrain A10 DA neuron stimulation—in GABAergic, dopaminergic, and glutamatergic systems across multiple neuronal hubs associated with depression. The study also examined the impact of stimulation temporality in the expression of the genetic markers. We report that mfb-DBS, both single/acute and repetitive/chronic, increased the expression of GABAergic markers, but not of either glutamatergic or of dopaminergic signalling. Interestingly, in the cases where unilateral stimulation affected the gene expression of the biomarker, the observed changes in the target regions were present on both the ipsilateral and the contralateral sides. This suggests the presence of collateral innervations that were also modulated by mfb-DBS to produce bilateral effects. Selective optogenetic stimulation of the dopaminergic pathways had no observable effects on the genetic markers of any of the three transmitter systems. The current finding that selective midbrain optogenetic stimulation of dopaminergic pathways did not induce changes in the expression of biomarkers of any of the three transmitter systems investigated suggests that transmitter release induction can be independent of gene expression changes.

The optogenetic stimulation parameters used (30 Hz/5 ms pulse width) were previously shown to have anti-depressant action^13,30^. The 5 ms pulse width provides sufficient time to activate neurons effectively while maintaining high temporal resolution, crucial for studying rapid neural dynamics^38,39^. The peak photocurrent increases with pulse width and saturates around 2 ms, suggesting that pulse widths slightly longer than this, such as 5 ms, can ensure maximal activation without unnecessary prolongation^40^.

Currently tested clinically, DBS is a promising experimental therapy for Treatment Resistant Depression patients in the numerous trials carried out over the last two decades^1,3,41,42^. The underlying mechanisms are not well understood, but multiple networks and neurochemical signalling pathways are thought to be implicated^43,44^. Mfb-DBS is non-selective as the electrical field discharged by the implanted electrodes will modulate—in a parameter dependent fashion—the multitude of bi-directional neurotransmitter pathways traversing the medial forebrain bundle^8,45^. DBS of the VTA in a chronic unpredictable mild stress mouse depression model has shown to both up- and downregulate the expression levels of genes linked to transcription factors, neurotransmitter receptors and transporters, and multiple other activity regulators such as mitogen-activated protein kinase^27,46^. The current study used two types of mfb-DBS parameters: low-frequency and long pulse width (30 Hz/5 ms) mirroring the optogenetic stimulation parameters; and high-frequency and short pulse width (130 Hz/100 µs) replicating the clinically relevant parameters. Increasing the pulse width from 100 µs to 30 ms means injecting more energy and thereby affecting a larger tissue volume in the mfb and activating more fibers. The frequency used also differentially affects the nearby neurones and fibers depending on their biophysical properties^47^.

Deficits in the GABAergic system observed in depression patients include reduced brain concentration of GABA, or changes in the subunit composition of GABAA receptors mediating GABAergic inhibition^48^. GABAA is a post-synaptic ionotropic receptor and ligand-gated ion channel with fast inhibitory effects. It is widely distributed in the CNS, including on the reward circuit^9,11^. GABAA activity has been shown to modulate anxiety- and “depression-like” behaviors^26^. It is not a direct marker of GABAergic neurons, but changes in the expression of *GABAA* (in the post-synaptic neuron) reflect overall activity of GABAergic neural transmission^49^. GAD1, on the other hand, is marker for GABAergic neurons: it is present in the presynaptic cleft, catalyzes the production of glutamate into GABA before its release^50^. GAD1 levels impact mood stability and the pathophysiology of affective disorders^51^.

In the present study, mfb-DBS resulted in the modulation of GABAergic signalling: *GABAA* expression increased in the mPFC and the LHb, and *GAD1* expression increased in the NAc, and the DStr. The majority of the GABAergic neurones are interneurons, but there are also GABAergic projections neurons^52,53^. The increase in GABAA in the mPFC and the LHb are likely related to mfb-DBS induced modulation of GABAergic interneuron activity. GABAergic interneurons are thought to be key regulators of gamma oscillations, and changes in gamma rhythms are now investigated as a potential biomarker for depression^54^. GABAergic interneuron activity is thought to be regulated by glutamatergic input^55^, and in the current study changes in GABAA expression in the mPFC can be explained via mfbDBS’s antidromic modulation of glutamatergic pathways connecting the mPFC and the midbrain. Less is known about the role of GABAergic interneurons in the LHb^56^, but increase in GABAA expression was likely affected by modulation by mfb-DBS of glutamatergic input into this structure. Finally, mfb-DBS increased *GAD1* expression in the DStr and the NAC via modulating inputs from the multiple fiber tracks traversing the mfb and projecting onto striatal targets. This likely signifies an increase in the activity of both the GABAergic striatal interneurons and the medial spiny GABAergic projection neurons, both offer important possible mechanisms that regulate major striatal output pathways.

Data from the study suggests the mfb-DBS neuromodulatory mechanisms activating GABAergic signalling are dopaminergic pathway independent, as the selective optogenetic activation of the midbrain VTA A10 mesocorticolimbic DA pathway did not alter neither *GABAA* nor *GAD1* gene expression in any of the monitored areas. Additional circumstantial evidence for dopamine-independent mechanisms is the absence of changes in *DAT* gene expression in midbrain A9 or A10 neurons. Similarly, no changes in the expression of the glutamatergic marker *Vglut2* were observed either, neither in the VTA nor in the LHb neurons under any stimulation conditions. The absence of quantifiable changes on the biomarkers associated with dopaminergic and glutamatergic transmission seem to be counterintuitive, and could be a misreading of the data due to the focus on a very limited number of biomarkers. Previous results, including from our group, shows that both mfb-DBS and optogenetic stimulation of midbrain A10 neurons result in dopamine release in striatal and PFC areas^14,35,57,58^. Taken together, this suggests that acute/single and chronic/repetitive stimulation can evoke dopamine release without accompanied modulation in the expression of the *DAT gene*. Furthermore, no significant impact on *Vglut2* expressing excitatory neurons in the VTA and LHb were observed either, implying that neither mfb-DBS nor optogenetic stimulation modulate the glutamatergic system in these regions under the specific experimental conditions applied. It is possible that under these particular stimulation conditions other biomarkers associated with dopaminergic (monoaminergic) transmission, for example, vesicular monoamine transporter—packaging dopamine presynaptically prior to release—might have shown change in expression on stimulation. Similarly, in the case of the glutamatergic transmission, tracing changes in excitatory amino acid transporters, might have shed different light on the mechanisms of neuromodulation as applied in the current study.

This study has several limitations. The experimental design did not permit to isolate the independent effect of pulse-width or frequency individually. The visualization/quantification of the changes in the targeted biomarker expressions was based on a coronal section (AP coordinate selected from the rat brain atlas^32^) that represented the largest cross-sectional area across the structure of interest. Although the coordinate was predefined and consistently applied across the analysis, quantification of greater samples would have produced more precise assessment of the changes in gene expression in the structures. In addition, choice of the biomarkers were selected based on their apparent importance, but, admittedly, in an arbitrarily way. Other relevant biomarkers probably would have generated different results. The change in gene expression of a selected marker is an indication of network/physiological activation, but not of functional activation. Furthermore, in-situ hybridization permits only semi-quantitative estimates of mRNA levels, and nuances between small, medium or large induced changes in gene expression are not easily detected.

In conclusion, our study suggests that both low and high frequency, acute/single and chronic/repetitive mfb-DBS activated gene expression of biomarkers associated with GABAergic transmission, but the increased expression were transitory and less chronic then predicted. The activations of GABAergic markers highlights their role in the regulation of mfb-DBS. The data suggests that network disinhibition—via modulation of the GABAergic signalling—could be a key mechanism of the anti-depressant therapeutic effects of clinical medial forebrain bundle DBS. On the other hand, selective optogenetic stimulation of midbrain dopaminergic pathways—although known to result in accumbal dopamine release—did not activate any of the tested biomarkers. This suggests that activation and release can occur without altering the expression of key biomarkers. Unilateral mfb-DBS had bilateral consequences on the gene expression likely via the activation of trans-commissural fibers. Bilateral effects of unilateral stimulation could inform clinical studies where stimulation protocols regularly use bilateral stimulation. Finally, a future study would need to include a rodent model of depression, expanded on the tested biomarkers associated with GABAergic, glutamatergic, and dopaminergic transmission, and assess physiological and behavioral read-outs.

## Electronic supplementary material

Below is the link to the electronic supplementary material.

Supplementary Material 1

Supplementary Material 2

## Data Availability

Data are provided within the manuscript or supplementary information files. All other raw data will be made available on request addressed to the Corresponding Author (Máté D Döbrössy, mate.dobrossy@uniklinikum-freiburg.de).

## References

[1] Herrman, H. et al. Time for united action on depression: A Lancet–World psychiatric association commission. *Lancet* **399**, 957–1022 (2022). 35180424 10.1016/S0140-6736(21)02141-3

[2] Schlaepfer, T. E., Bewernick, B. H., Kayser, S., Mädler, B. & Coenen, V. A. Rapid effects of deep brain stimulation for treatment-resistant major depression. *Biol. Psychiatry* **73**, 1204–1212 (2013). 23562618 10.1016/j.biopsych.2013.01.034

[3] Bewernick, B. H. et al. Deep brain stimulation of the supero-lateral branch of the medial forebrain bundle does not lead to changes in personality in patients suffering from severe depression. *Psychol. Med.* 1–9. 10.1017/S0033291718000296 (2018). 10.1017/S0033291718000296 29493478

[4] Coenen, V. A. et al. The heart asks pleasure first’-conceptualizing psychiatric diseases as MAINTENANCE network dysfunctions through insights from SlMFB DBS in depression and obsessive-compulsive disorder. *Brain Sci.* **12**, 438 (2022). 35447971 10.3390/brainsci12040438 PMC9028695

[5] Fenoy, A. J., Quevedo, J. & Soares, J. C. Deep brain stimulation of the ‘medial forebrain bundle’: A strategy to modulate the reward system and manage treatment-resistant depression. *Mol. Psychiatry* **27**, 574–592 (2022). 33903731 10.1038/s41380-021-01100-6

[6] Thiele, S., Furlanetti, L., Pfeiffer, L. M., Coenen, V. A. & Döbrössy, M. D. The effects of bilateral, continuous, and chronic deep brain stimulation of the medial forebrain bundle in a rodent model of depression. *Exp. Neurol.* **303**, 153–161 (2018). 29428214 10.1016/j.expneurol.2018.02.002

[7] Thiele, S. et al. Deep brain stimulation of the medial forebrain bundle in a rodent model of depression: Exploring dopaminergic mechanisms with raclopride and micro-PET. *Stereotact. Funct. Neurosurg.* **98**, 8–20 (2020). 31982883 10.1159/000504860

[8] Döbrössy, M. D. et al. Neuromodulation in psychiatric disorders: Experimental and clinical evidence for reward and motivation network deep brain stimulation: Focus on the medial forebrain bundle. *Eur. J. Neurosci.* **53**, 89–113 (2021). 32931064 10.1111/ejn.14975

[9] Russo, S. J. & Nestler, E. J. The brain reward circuitry in mood disorders. *Nat. Rev. Neurosci.* **14**, 609–625 (2013). 23942470 10.1038/nrn3381 PMC3867253

[10] Alcaro, A. & Panksepp, J. The SEEKING mind: Primal neuro-affective substrates for appetitive incentive states and their pathological dynamics in addictions and depression. *Neurosci. Biobehav Rev.* **35**, 1805–1820 (2011). 21396397 10.1016/j.neubiorev.2011.03.002

[11] Heshmati, M. & Russo, S. J. Anhedonia and the brain reward circuitry in depression. *Curr. Behav. Neurosci. Rep.* **2**, 146–153 (2015). 26525751 10.1007/s40473-015-0044-3 PMC4626008

[12] Jun-Li, C. et al. Mesolimbic dopamine neurons in the brain reward circuit mediate susceptibility to social defeat and antidepressant action. *J. Neurosci.* **30**, 16453 (2010). 21147984 10.1523/JNEUROSCI.3177-10.2010 PMC3061337

[13] Tong, Y., Pfeiffer, L., Serchov, T., Coenen, V. A. & Döbrössy, M. D. Optogenetic stimulation of ventral tegmental area dopaminergic neurons in a female rodent model of depression: The effect of different stimulation patterns. *J. Neurosci. Res.* **100**, 897–911 (2022). 35088434 10.1002/jnr.25014

[14] Ashouri Vajari, D. et al. Medial forebrain bundle DBS differentially modulates dopamine release in the nucleus accumbens in a rodent model of depression. *Exp. Neurol.* **327**, 113224–113224 (2020). 32035070 10.1016/j.expneurol.2020.113224

[15] Leandra, R. M. et al. Defensive behaviors driven by a hypothalamic-ventral midbrain circuit. *Eneuro* **6**, ENEURO.0156-19.2019 (2019). 10.1523/ENEURO.0156-19.2019 PMC6664144 31331938

[16] Yu, X. et al. GABA and glutamate neurons in the VTA regulate sleep and wakefulness. *Nat. Neurosci.* **22**, 106–119 (2019). 30559475 10.1038/s41593-018-0288-9 PMC6390936

[17] Barbano, M. F. et al. VTA glutamatergic neurons mediate innate defensive behaviors. *Neuron* **107**, 368–382e8 (2020). 32442399 10.1016/j.neuron.2020.04.024 PMC7381361

[18] Zell, V. et al. VTA glutamate neuron activity drives positive reinforcement absent dopamine Co-release. *Neuron* **107**, 864–873e4 (2020). 32610039 10.1016/j.neuron.2020.06.011 PMC7780844

[19] Stuber, G. D., Hnasko, T. S., Britt, J. P., Edwards, R. H. & Bonci, A. Dopaminergic terminals in the nucleus accumbens but not the dorsal striatum corelease glutamate. *J. Neurosci.* **30**, 8229 (2010). 20554874 10.1523/JNEUROSCI.1754-10.2010 PMC2918390

[20] Fatuel, T. et al. Glutamatergic signaling by mesolimbic dopamine neurons in the nucleus accumbens. *J. Neurosci.* **30**, 7105 (2010). 20484653 10.1523/JNEUROSCI.0265-10.2010 PMC3842465

[21] Yamaguchi, T., Wang, H. L., Li, X., Ng, T. H. & Morales, M. Mesocorticolimbic glutamatergic pathway. *J. Neurosci.* **31**, 8476 (2011). 21653852 10.1523/JNEUROSCI.1598-11.2011 PMC6623324

[22] Gorelova, N., Mulholland, P. J., Chandler, L. J. & Seamans, J. K. The glutamatergic component of the mesocortical pathway emanating from different subregions of the ventral midbrain. *Cereb. Cortex* **22**, 327–336 (2012). 21666135 10.1093/cercor/bhr107 PMC3256405

[23] Hnasko, T. S. et al. Vesicular glutamate transport promotes dopamine storage and glutamate corelease in vivo. *Neuron* **65**, 643–656 (2010). 20223200 10.1016/j.neuron.2010.02.012 PMC2846457

[24] Morales, M. & Root, D. H. Glutamate neurons within the midbrain dopamine regions. *Ventral Tegmentum Dopamine New. Wave Divers.* **282**, 60–68 (2014). 10.1016/j.neuroscience.2014.05.032 PMC4397110 24875175

[25] Yang, Y. et al. Ketamine blocks bursting in the lateral Habenula to rapidly relieve depression. *Nature* **554**, 317–322 (2018). 29446381 10.1038/nature25509

[26] Luscher, B., Maguire, J. L., Rudolph, U. & Sibille, E. GABAA receptors as targets for treating affective and cognitive symptoms of depression. *Trends Pharmacol. Sci.* **44**, 586–600 (2023). 37543478 10.1016/j.tips.2023.06.009 PMC10511219

[27] Song, N. et al. NAc-DBS corrects depression-like behaviors in CUMS mouse model via disinhibition of DA neurons in the VTA. *Mol. Psychiatry* **29**, 1550–1566 (2024). 38361128 10.1038/s41380-024-02476-x

[28] Zimmermann, M. Ethical guidelines for investigations of experimental pain in conscious animals. *Pain* **16**, 109–110 (1983). 6877845 10.1016/0304-3959(83)90201-4

[29] Kilkenny, C., Browne, W. J., Cuthill, I. C., Emerson, M. & Altman, D. G. Improving bioscience research reporting: The ARRIVE guidelines for reporting animal research. *PLoS Biol.* **8**, e1000412 (2010). 20613859 10.1371/journal.pbio.1000412 PMC2893951

[30] Tye, K. M. et al. Dopamine neurons modulate neural encoding and expression of depression-related behaviour. *Nature* **493**, 537–541 (2013). 23235822 10.1038/nature11740 PMC4160519

[31] Furlanetti, L. L., Coenen, V. A., Aranda, I. A. & Döbrössy, M. D. Chronic deep brain stimulation of the medial forebrain bundle reverses depressive-like behavior in a Hemiparkinsonian rodent model. *Exp. Brain Res.*10.1007/s00221-015-4375-9 (2015). 26195164 10.1007/s00221-015-4375-9 PMC4623086

[32] Paxinos, G. & Watson, C. *The Rat Brain in Stereotaxic Coordinates* (Academic, 2007). 10.1016/0165-0270(80)90021-7 6110810

[33] Nieuwenhuys, R., Geeraedts, L. M. G. & Veening, J. G. The medial forebrain bundle of the rat. I. General introduction. *J. Comp. Neurol.* **206**, 49–81 (1982). 6124562 10.1002/cne.902060106

[34] Miguel Telega, L., Ashouri Vajari, D., Ramanathan, C., Coenen, V. A. & Döbrössy, M. D. Chronic in vivo sequelae of repetitive acute mfb-DBS on accumbal dopamine and midbrain neuronal activity. *J. Neurochem*. 10.1111/jnc.16223 (2024). 39308085 10.1111/jnc.16223 PMC11658194

[35] Miguel Telega, L., Ashouri Vajari, D., Stieglitz, T., Coenen, V. A. & Döbrössy, M. D. New insights into in vivo dopamine physiology and neurostimulation: A fiber photometry study highlighting the impact of medial forebrain bundle deep brain stimulation on the nucleus accumbens. *Brain Sci.* **12** (2022). 10.3390/brainsci12081105 PMC9406226 36009169

[36] Patriarchi, T. et al. An expanded palette of dopamine sensors for multiplex imaging in vivo. *Nat. Methods*. **17**, 1147–1155 (2020). 32895537 10.1038/s41592-020-0936-3 PMC8169200

[37] Settell, M. L. et al. Functional circuitry effect of ventral tegmental area deep brain stimulation: Imaging and neurochemical evidence of mesocortical and mesolimbic pathway modulation. *Front. Neurosci.* **11**, 104 (2017). 28316564 10.3389/fnins.2017.00104 PMC5334355

[38] Adamantidis, A. R. et al. Optogenetic interrogation of dopaminergic modulation of the multiple phases of reward-seeking behavior. *J. Neurosci. Off J. Soc. Neurosci.* **31**, 10829–10835 (2011). 10.1523/JNEUROSCI.2246-11.2011 PMC3171183 21795535

[39] Hughes, R. N. et al. Ventral tegmental dopamine neurons control the impulse vector during motivated behavior. *Curr. Biol. CB*. **30**, 2681–2694e5 (2020). 32470362 10.1016/j.cub.2020.05.003 PMC7590264

[40] Boyden, E. S., Zhang, F., Bamberg, E., Nagel, G. & Deisseroth, K. Millisecond-timescale, genetically targeted optical control of neural activity. *Nat. Neurosci.* **8**, 1263–1268 (2005). 16116447 10.1038/nn1525

[41] Roet, M. et al. Deep brain stimulation for treatment-Resistant depression: Towards a more personalized treatment approach. *J. Clin. Med.* **9**, 2729 (2020). 32846987 10.3390/jcm9092729 PMC7565181

[42] Figee, M. et al. Deep brain stimulation for depression. *Neurother. J. Am. Soc. Exp. Neurother.* **19**, 1229–1245 (2022). 10.1007/s13311-022-01270-3 PMC9587188 35817944

[43] Herrington, T. M., Cheng, J. J. & Eskandar, E. N. Mechanisms of deep brain stimulation. *J. Neurophysiol.* **115**, 19–38 (2016). 26510756 10.1152/jn.00281.2015 PMC4760496

[44] Höflich, A., Michenthaler, P., Kasper, S. & Lanzenberger, R. Circuit mechanisms of reward, anhedonia, and depression. *Int. J. Neuropsychopharmacol.* **22**, 105–118 (2019). 30239748 10.1093/ijnp/pyy081 PMC6368373

[45] Döbrössy, M. D., Furlanetti, L. L. & Coenen, V. A. Electrical stimulation of the medial forebrain bundle in pre-clinical studies of psychiatric disorders. *Neurosci. Biobehav Rev.* **49**, 32–42 (2015). 25498857 10.1016/j.neubiorev.2014.11.018

[46] Song, N., Du, J., Gao, Y. & Yang, S. Epitranscriptome of the ventral tegmental area in a deep brain-stimulated chronic unpredictable mild stress mouse model. *Transl Neurosci.* **11**, 402–418 (2020). 33343932 10.1515/tnsci-2020-0146 PMC7724003

[47] Ng, P. R., Bush, A., Vissani, M., McIntyre, C. C. & Richardson, R. M. Biophysical principles and computational modeling of deep brain stimulation. *Neuromodulation J. Int. Neuromodulation Soc.* **27**, 422–439 (2024). 10.1016/j.neurom.2023.04.471 37204360

[48] Luscher, B., Shen, Q. & Sahir, N. The GABAergic deficit hypothesis of major depressive disorder. *Mol. Psychiatry* **16**, 383–406 (2011). 21079608 10.1038/mp.2010.120 PMC3412149

[49] Barnes, E. M. Use-dependent regulation of GABAA receptors. *Int. Rev. Neurobiol.* **39**, 53–76 (1996). 8894844 10.1016/s0074-7742(08)60663-7

[50] Langendorf, C. G. et al. Structural characterization of the mechanism through which human glutamic acid decarboxylase auto-activates. *Biosci. Rep.* **33**, 137–144 (2013). 23126365 10.1042/BSR20120111 PMC3546353

[51] Sanacora, G., Treccani, G. & Popoli, M. Towards a glutamate hypothesis of depression: An emerging frontier of neuropsychopharmacology for mood disorders. *Neuropharmacology* **62**, 63–77 (2012). 21827775 10.1016/j.neuropharm.2011.07.036 PMC3205453

[52] Caputi, A., Melzer, S., Michael, M. & Monyer, H. The long and short of GABAergic neurons. *Curr. Opin. Neurobiol.* **23**, 179–186 (2013). 23394773 10.1016/j.conb.2013.01.021

[53] Melzer, S. & Monyer, H. Diversity and function of corticopetal and corticofugal GABAergic projection neurons. *Nat. Rev. Neurosci.* **21**, 499–515 (2020). 32747763 10.1038/s41583-020-0344-9

[54] Fitzgerald, P. J. & Watson, B. O. Gamma oscillations as a biomarker for major depression: An emerging topic. *Transl Psychiatry* **8**, 177 (2018). 30181587 10.1038/s41398-018-0239-y PMC6123432

[55] Carlén, M. et al. A critical role for NMDA receptors in parvalbumin interneurons for gamma rhythm induction and behavior. *Mol. Psychiatry* **17**, 537–548 (2012). 21468034 10.1038/mp.2011.31 PMC3335079

[56] Zhang, L. et al. A GABAergic cell type in the lateral Habenula links hypothalamic homeostatic and midbrain motivation circuits with sex steroid signaling. *Transl Psychiatry* **8**, 50 (2018). 29479060 10.1038/s41398-018-0099-5 PMC5865187

[57] Chaudhury, D. et al. Rapid regulation of depression-related behaviours by control of midbrain dopamine neurons. *Nature* **493**, 532–536 (2013). 23235832 10.1038/nature11713 PMC3554860

[58] Pallikaras, V. & Shizgal, P. The convergence model of brain reward circuitry: Implications for relief of treatment-resistant depression by deep-brain stimulation of the medial forebrain bundle. *Front. Behav. Neurosci.* **16** (2022). 10.3389/fnbeh.2022.851067 PMC9011331 35431828

