# Supplementary figures and images for: Acute and chronic gene expression activation following medial forebrain bundle DBS and selective dopamine pathway stimulation
Source: Sci Rep. 2025 Feb 28;15:7131. doi: 10.1038/s41598-025-91994-x (PMC11871370; doi:10.1038/s41598-025-91994-x)

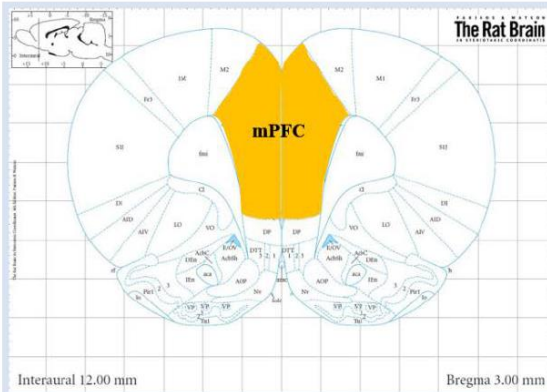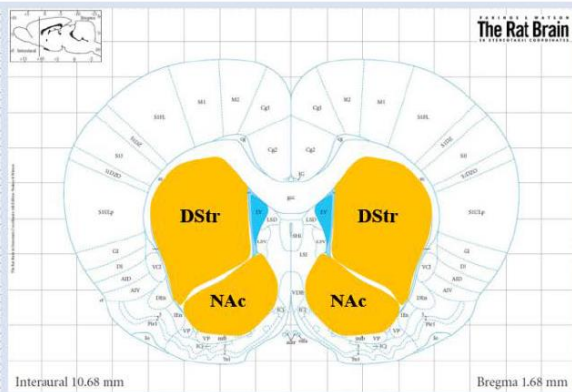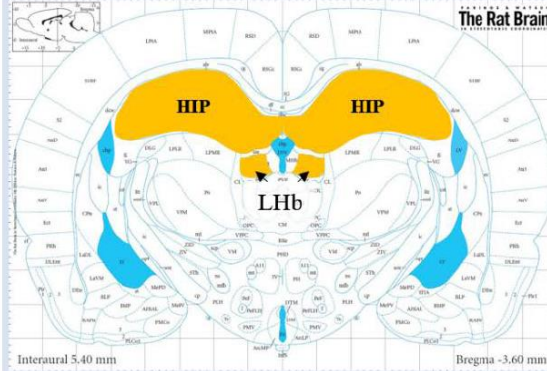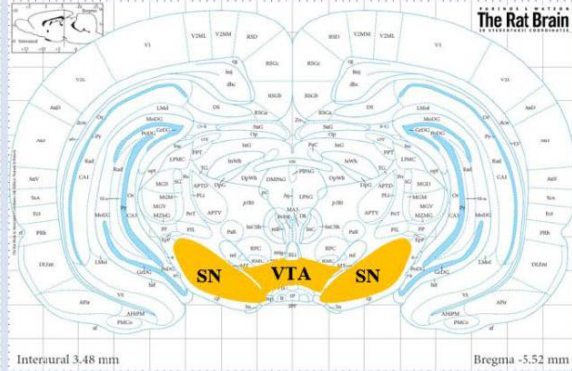

Supplement: Supplementary file 1 — Supplementary Material 1 [file 41598_2025_91994_MOESM1_ESM.pdf]
