# Supplementary material for: Acute and chronic gene expression activation following medial forebrain bundle DBS and selective dopamine pathway stimulation
Source: Sci Rep. 2025 Feb 28;15:7131. doi: 10.1038/s41598-025-91994-x (PMC11871370; doi:10.1038/s41598-025-91994-x)
Supplement: Supplementary file 2 — Supplementary Material 2 [file 41598_2025_91994_MOESM2_ESM.pdf]

RNA probes for DAT, VGLUT2, and GABAA, GAD1

1. DAT

```
   1 ggccagacca agagggaaga agcacagaat tcctcaactc ccagtgtgcc catgagtaag
  61 agcaaatgct ccgtgggact catgtcttcc gtggtggccc cggctaagga gcccaatgcc
 121 gtgggcccga aggaggtgga gctcatcctt gtcaaggagc agaacggagt gcagctcacc
 181 agctccaccc tcaccaaccc gcggcagagc cccgtggagg cccaggatcg ggagacctgg
 241 ggcaagaaga tcgactttct cctgtccgtc attggctttg ctgtggacct ggccaacgtc
 301 tggcggttcc cctacctgtg ctacaaaaat ggtggcggtg ccttcctggt cccctacctg
 361 ctcttcatgg tcattgctgg gatgccactt ttctacatgg agctggccct cggccagttc
 421 aacagggaag gggccgctgg tgtctggaag atctgcccca tactgaaagg tgtgggcttc
 481 acggtcatcc tcatctcact gtatgtcggc ttcttctaca acgtcatcat cgcctgggcg
 541 ctgcactatc tcttctcctc cttcaccacg gagctcccct ggatccactg caacaactcc
 601 tggaacagcc ccaactgctc ggatgcccat cctggtgact ccagtggaga cagctcgggc
 661 ctcaacgaca cttttgggac cacacctgct gccgagtact ttgaacgtgg cgtgctgcac
 721 ctccaccaga gccatggcat cgacgacctg gggcctccgc ggtggcagct cacagcctgc
 781 ctggtgctgg tcatcgtgct gctctacttc agcctctgga agggcgtgaa gacctcaggg
 841 aaggtggtat ggatcacagc caccatgcca tacgtggtcc tcactgccct gctcctgcgt
 901 ggggtcaccc tccctggagc catagacggc atcagagcat acctgagcgt tgacttctac
 961 cggctctgcg aggcgtctgt ttggattgac gcggccaccc aggtgtgctt ctccctgggc
1021 gtggggttcg ggtgctgat  cgccttctcc agctacaaca agttcaccaa caactgctac
1081 agggacgcga ttgtcaccac ctccatcaac tccctgacga gcttctcctc cggcttcgtc
1141 gtcttctcct tcctggggta catggcacag aagcacagtg tgcccatcgg ggacgtggcc
1201 aaggacgggc cagggctgat cttcatcatc tacccggaag ccatcgccac gctccctctg
1261 tcctcagcct gggccgtggt cttcttcatc atgctgctca ccctgggtat cgacagcgcc
1321 atgggtggta tggagtcagt gatcaccggg ctcatcgatg agttccagct gctgcacaga
1381 caccgtgagc tcttcacgct cttcatcgtc ctggcgacct tcctcctgtc cctgttctgc
1441 gtcaccaacg gtggcatcta cgtcttcacg ctcctggacc attttgcagc cggcacgtcc
1501 atcctctttg gagtgctcat cgaagccatc ggagtggcct ggttctatgg tgttgggcag
1561 ttcagcgacg acatccagca gatgaccggg cagcggccca gcctgtactg gcggctgtgc
1621 tggaagctgg tcagcccctg ctttctcctg ttcgtggtcg tggtcagcat tgtgaccttc
1681 agaccccccc actacggagc ctacatcttc cccgactggg ccaacgcgct gggctgggtc
1741 atcgccacat cctccatggc catggtgccc atctatgcgg cctacaagtt ctgcagcctg
1801 cctgggtcct ttcgagagaa actggcctac gccattgcac ccgagaagga ccgtgagctg
1861 gtggacagag gggaggtgcg ccagttcacg ctccgccact ggctcaaggt gtagagggag
1921 cagagacgaa gaccccagga agtcatcctg caatgggaga gacacgaaca aaccaaggaa
1981 atctaagttt cgagagaaag gagggcaact tctactcttc aacctctact gaaaacacaa
2041 acaacaaagc agaagactcc tctcttctga ctgtttacac ctttccgtgc cgggagcgca
2101 cctcgccgtg tcttgtgttg ctgtaataac gacgtagatc tgtgcagcga ggtccacccc
2161 gttgttgtcc ctgcagggca gaaaaacgtc taacttcatg ctgtctgtgt gaggctccct
2221 ccctccctgc tccctgctcc cggctctgag gctgccccag gggcactgtg ttctcaggcg
2281 gggatcacga tccttgtaga cgcacctgct gagaatcccc gtgctcacag tagcttccta
2341 gaccatttac tttgcccata ttaaaaagcc aagtgtcctg cttggtttag ctgtgcagaa
2401 ggtgaaatgg aggaaaccac aaattcatgc aaagtccttt cccgatgcgt ggctcccagc
2461 agaggccgta aattgagcgt tcagttgaca cattgcacac acagtctgtt cagaggcatt
2521 ggaggatggg ggtcctggta tgtctcacca ggaaattctg tttatgttct tgcagcagag
2581 agaaataaaa ctccttgaaa ccagctcagg ctactgccac tcaggcagcc tgtgggtcct
2641 tgcggtgtag ggaacggcct gagaggagcg tgtcctatcc ccggacgcat gcagggcccc
2701 cacaggagcg tgtcctatcc ccggacgcat gcagggcccc cacaggagcg tgtactaccc
2761 cagaacgcat gcag
```

2. VGLUT2

```
   1 agctattctg ttgtactctc tttctcgccc tccctcccct ctccaactca cagccttgct
  61 ggaaagctca cctctgctga gaagaaaaag ctctacctta accaactaag actatgcgca
 121 gaatccgtct ttcatagcca caacaattta aatctggtaa ggctggacac cagtctttac
 181 aagaatggag tcggtaaaac aaaggatttt ggccccgggg aaagagggga taaagaattt
 241 tgctggaaaa tccctcggac agatctacag ggtgctggag aagaagcagg acaaccgaga
 301 gaccatcgag ctgacagagg acggtaagcc cctggaggtg cctgagaaga aggctccgct
 361 atgcgactgc acgtgcttcg gcctgccgcg ccgctacatc atagccatca tgagcggcct
 421 cggcttctgc atatccttcg gcatccgctg taacctgggc gtggccatcg tggacatggt
 481 caacaacagc actatccacc gcggaggcaa agttatcaag gagaaagcca aatttaactg
 541 ggaccccgag accgtgggga tgatccacgg atcgttcttc tggggctata tcatcaccca
 601 gattccagga ggatatatcg catcgcggct ggctgctaac cgggtctttg gggctgcgat
 661 actgctcacc tctaccctca atatgctgat cccatctgca gccagagtgc attatggatg
 721 tgtcatcttt gttaggatat tgcaaggact tgtggagggt gtcacctacc cagcctgtca
 781 tgggatatgg agcaagtggg cccctccctt ggagaggagt aggttggcta caacctcctt
 841 ttgtggttcc tatgctggag cagtcattgc aatgccctta gctggtatcc ttgtgcagta
 901 cactggatgg tcgtcagtat tttatgtgta tggaagcttt ggcatggtct ggtacatgtt
 961 ctggcttctg gtgtcttatg agagccctgc aaagcatcct accattacag atgaagaacg
1021 taggtacata gaggagagca ttggagagag cgcaaatctg ctaggtgcaa tggaaaaatt
1081 taagacccca tggaggaagt tttcacatc catgcccgtc tacgcgataa ttgttgccaa
1141 cttctgcagg agctggactt tttatttact gctcatcagt cagccagctt attttgagga
1201 ggtttttgga tttgaaatca gcaaggttgg catgttgtct gcagtccctc accttgtcat
1261 gacaatcatt gtgcctatcg gggggcaaat tgcagatttc ctaaggagca agcaaattct
1321 ctcaacaact acagtgagaa agatcatgaa ttgtgggggt tttggcatgg aagccacgct
1381 gcttctggtt gttggctact ctcatactag aggggtggcc atctccttct tggtgcttgc
1441 agtaggattc agtggatttg ctatctctgg tttcaatgtt aatcacttgg atattgctcc
1501 aagatatgcc agtatcttaa tgggcatttc aaatggcgtt ggcacgctgt cggggatggt
1561 ttgccctatc attgttggtg caatgacaaa gaataagtcc cgtgaagaat ggcagtatgt
1621 cttcctcatt gctgcactcg tccactatgg tggagtcata ttttatgcac tatttgcctc
1681 aggagagaaa caaccttggg cagaccctga ggaaacaagc gaagaaaaat gtggcttcat
1741 tcacgaagat gaactggatg aagaaacggg ggacatcact cagaattaca taaattacgg
1801 taccaccaaa tcttacggtg ctacctcaca ggagaatgga ggctggccta acggctggga
1861 gaaaaaggaa gaatttgtgc aagaaggtgc gcaagacgcg tacacctata aggaccgaga
1921 tgattattca taacgatgct agttgctgga ttcatttgta gtgtttgtga atcaattaat
1981 tgtgattgca caaaaataat tttaaaaatg tggtgtgaac atgtaaacat atcaaccaag
2041 caagtcttgc tgttcaaaaa caaaaacaaa aaaatctgaa ttcaaaacag accatgagat
2101 tcccatcaag tgcaatctgt ggcagttgtc acgttatgcc gtcttcattc aggccatttg
2161 tcctttcgtt tgtgatttaa aggtttcctg tagaaataag taggtattcg ttggacccat
2221 caccatttta gagagcacaa ctacaacagt tggcacatgt catcctacag aagttaggaa
2281 gccaaagcta ctggatcatg caaactgcac ttatttatta cactggactg caaactatcc
2341 cagggaaagc ctgtctagag acatagtgga acaggaaaga tggctagatc aggtattgac
2401 tataatcatt atgtgtctat catggagtgg ctatatcttt caatgaagaa ctatattgtg
2461 tagctagcaa actgtactga actcttacta ggagtgcaca gtgtgtgata ttttgtgatc
2521 ttccaaaagc ttatcttgca gtgttttgtg aaatgcttgg gcacaaacac ttatttttat
2581 gaatgagagc ttgtaaaggg aggggtatgc tccatgctcc cccattcact acctgacagt
2641 atcaaaccct tcacatttca ataaaatcca actttcatgt aacatatcac ataacttttt
2701 tttttgcaa aaaaataaga agaaatagac ttcaatgtat tttttattac aactttgtac
2761 tggttgtaac ttgcattagg aaaaaatgat taatatatgt ataatcataa agaatctaat
2821 aaaaatttac tatgatgata tagcccttaa aatgcaatat taacaaaata aatagaaaat
2881 ttagataatc ttccttgata actagagact atatgaaact catgccacaa agctatatat
2941 aatatgaaga gataaacaat agagattcta tatgtagaca attttattac ctaatgtccc
3001 acataagaga tatttgtctt gagtatatag tacaaaatat attaaaatta tatctacatc
3061 cctgtatatc ttatacatat ccactcacac gaacataaca aatacttttc acacagaacc
3121 aaaaacaagc atacacctaa tgttgggttt ggggattgca atttctactt tcatagagtc
3181 atagaatttt agatggaaaa aaaaaagcat tttgctcgtc atttcttaat ataattcaac
3241 aggaactgca acatttgtgt accaagcaat aagtgcaaag cataaaacct cctgtgtgta
3301 aattatcccc atgttgcttg tggtagcagt gatttttttt ttttaaaaaa gttaacctca
3361 gagctctttt taatgttttg cgctgataag aatgcacatc ccaatttact gcaaaatgtc
3421 acctggtgtg tttacctgtc agttttgggt atttgatctg tctggtgtcc tgtgctcttg
3481 actggaggcc ctgctactgc aaatatacaa tgtgaagttt gtttttaaat gcaaaccact
3541 cctgacctta agaaactgaa gtccctctct gctttgtgtc tcagagtact atcatgtgac
3601 cataaccttt gctgtgctga gtaaagagat gtgaactgtc attttgttgc tgcaaaacaa
3661 gtgttaataa aatgttcttt tttaaaaaaa aaaaaaaaaa aaaaaaaaaa aaaaaaaaaa
3721 aaaaaaaa
```

3. GABAA

```
  1 gggaagcaaa tttgggtatg aaatctctag tgcaggagca cgcagagtcc atgatggctc
 61 aaaccgtgtg agtgagcgcg gcgcgaggac gccctccgcc cggcgcgccc gcgctcgcac
121 actcgcgcag ctccggctca ccgcgatcct ctctcccaca cttttctccc gggtctggag
181 cgatccggtg cccagagggg gccccgagct ggacaagccc gtgatgaaga aaagtcgggg
241 tctctctgac tatctttggg cctggaccct cattctgagc actctctcgg gaagaagcta
301 tggacagccc tcccaagatg aacttaagga caacaccact gtcttcacga ggattttgga
361 ccgactgctg gatggttatg acaatcgtct gagaccaggc ttgggagagc gtgtaactga
421 agtgaagacg gacatctttg tcaccagttt cggacccgtg tcagaccacg atatggaata
481 tacaatagat gtgtttttcc gccaaagctg gaaggatgaa agattaaaat tcaaaggacc
541 catgacagtg ctccggctga acaacctgat ggccagtaaa atctggactc cagatacatt
601 tttccacaat ggaaaaaagt ctgtggccca caacatgacc atgcccaata aactcctgcg
661 tatcacagag gatggcacac tgctgtacac catgaggttg actgtgagag ccgatgcccc
721 atgcacttag aagactttcc atggattgcc atgctgccac taaaattggg agctatgcta
781 t
```

4. GAD1

```
  1 gtaagcagcc ctggggtgac acccatcacg tactcctgtt gacagagccg agtcccagcc
 61 cagccccgtg gacgcttcgc agaggagtcg cggtgagggg tccagctcgc tgtcgctgaa
121 ccgagcctgt tcctgcgccc agtctgcggg ggaccctttg aaccgtagag accccaagac
181 caccgagctg atggcatctt ccactccttc gcctgcaacc tcctcgaacg cgggagcggg
241 atcctaatac atagcaacct gcgccctaca acgttatgat acttgtgtgt ggcgtagccc
301 caatggatgc accagaaaac tgggcctgca agatctgtgg cattcgttac aaacggacca
361 atagcctgga aggagaagag tcgtcttgtg agcgccttca gggagaggca gtccctccaa
421 agaaccttgc tttcctgtga taaccgttga ccagggtgcc cgacttccgg aggcacagag
481 accgacttct ccaaacctgt ttgcctcaag atctgcttac caggcctaac gaacggggag
541 gagcaaaact gcgcagcttc ttgctcggaa gtgggtagac aatactcctt cacacttatt
601 gtccgacagg aacacttttg attcgctcca gccacagggt actggaattt caagcgacgc
661 cacacaccgt ttgctggaag agccattgca caagggcttt cactgtggaa gctgtgcttg
721 acccaccccg acgttcatct ggcatgcagg aatcctggct agacggtcga cgcaccc
```
